# Annonaceous acetogenin mimic AA005 induces cancer cell death via apoptosis inducing factor through a caspase-3-independent mechanism

**DOI:** 10.1186/s12885-015-1133-0

**Published:** 2015-03-18

**Authors:** Bing Han, Tong-Dan Wang, Shao-Ming Shen, Yun Yu, Chan Mao, Zhu-Jun Yao, Li-Shun Wang

**Affiliations:** 1 Center for Molecular Medicine, Ruijin Hospital, Shanghai Jiao Tong University School of Medicine, Shanghai, 200025 China; 2 State Key Laboratory of Coordination Chemistry, Institute of Chemical Biology and Drug Innovation, School of Chemistry and Chemical Engineering, Nanjing University, Nanjing, 210093 P. R. China; 3 Shanghai Universities E-Institute for Chemical Biology, Shanghai, 200025 China

**Keywords:** Annonaceous acetogenins, Cancer, AIF, ROS, RIP-1

## Abstract

**Background:**

Annonaceous acetogenins are a family of natural products with antitumor activities. Annonaceous acetogenin mimic AA005 reportedly inhibits mammalian mitochondrial NADH-ubiquinone reductase (Complex I) and induces gastric cancer cell death. However, the mechanisms underlying its cell-death-inducing activity are unclear.

**Methods:**

We used SW620 colorectal adenocarcinoma cells to study AA005 cytotoxic activity. Cell deaths were determined by Trypan blue assay and flow cytometry, and related proteins were characterized by western blot. Immunofluorescence and subcellular fractionation were used to evaluate AIF nuclear translocation. Reactive oxygen species were assessed by using redox-sensitive dye DCFDA.

**Results:**

AA005 induces a unique type of cell death in colorectal adenocarcinoma cells, characterized by lack of caspase-3 activation or apoptotic body formation, sensitivity to poly (ADP-ribose) polymerase inhibitor Olaparib (AZD2281) but not pan-caspase inhibitor Z-VAD.fmk, and dependence on apoptosis-inducing factor (AIF). AA005 treatment also reduced expression of mitochondrial Complex I components, and leads to accumulation of intracellular reactive oxygen species (ROS) at the early stage. Blocking ROS formation significantly suppresses AA005-induced cell death in SW620 cells. Moreover, blocking activation of RIP-1 by necroptosis inhibitor necrotatin-1 inhibits AIF translocation and partially suppresses AA005-induced cell death in SW620 cells demonstrating that RIP-1 protein may be essential for cell death.

**Conclusions:**

AA005 may trigger the cell death via mediated by AIF through caspase-3 independent pathway. Our work provided new mechanisms for AA005-induced cancer cell death and novel clues for cancer treatment via AIF dependent cell death.

## Background

Biochemical qualities of the *Annonaceae* (custard-apple) family are not completely known due to its large size (130 genera and 2300 species) [1]. Many *Annonaceae* species have been used in folk medicine and as insecticides [2]. Products from the *Annonaceae* family, collectively called annonaceous acetogenins (AAs), are very potent inhibitors of mammalian mitochondria NADH-ubiquinone reductase (Complex I) [3]. To date, over 400 members of this compound family have been found, most of which have been proven to exhibit high cytotoxic and antitumor activities [1]. Over the past few years, we have successfully developed a series of AA mimetics. More interestingly, we found that some of these analogues have significant selectivity between human cancer cells and normal cells [4]. AA005 shows the best inhibitory effect against several human cancer cell lines [5], although its exact mechanisms are largely unknown.

Mitochondria are the central relay station for apoptotic signal transduction. In response to apoptotic stimulus, permeabilized mitochondria release cytochrome c into the cytoplasm, where cytochrome c forms an apoptosome with Apaf-1 and caspase-9 and triggers the caspase cascade. The most important caspase in this cascade is caspase-3, which is cleaved and activated to transduce the apoptotic signal [6,7]. Mitochondria can also release apoptosis-inducing factor (AIF) to initiate caspase-independent cell death [8,9]. The mitochondrial flavoprotein AIF is a caspase-independent cell-death-inducing factor [10]. During apoptotic signaling without caspase-3 activation, AIF is released from the mitochondria when the mitochondrial membrane is permeabilized, then translocates to the nucleus where it induces cell death by triggering chromatin condensation and large-scale DNA fragmentation into ~50-kilobase strands with the help of other proteins such as Endo G (*C. elegans*), CypA (mice) or FEN-1 [10-17]. Here we report that AA005 may trigger caspase-3-independent cell death, mediated by AIF. Our work may provide novel therapeutic clues for treating cancers via a non-canonical apoptotic pathway.

## Methods

### Cell culture and treatments

Human colorectal adenocarcinoma cell line SW620, breast cancer cell line BT-549, and U937 acute myelomonocytic leukemic cell line came from the Cell Bank of Shanghai Institutes for Biological Sciences (Shanghai, China); acute promyelocytic leukemia (APL) cell line NB4 were kindly provided by Dr. M. Lanotte in France [18]. These cells were cultured in RPMI-1640 medium (Sigma-Aldrich, St Louis, MO) supplemented with 10% heat-inactivated fetal calf serum (FCS; HyClone, Logan, UT) in a 5% CO_2_ humidified atmosphere at 37°C. For experiments, cells were seeded at 2–5 × 10^5^ cells/ml and incubated with the indicated concentrations of AA mimic AA005 (kindly provided by Institute of Chemical Biology and Drug Innovation, School of Chemistry and Chemical Engineering, Nanjing University, Nanjing, China), MNNG (Sigma-Aldrich), and camptothecin (kindly provided by National Cancer Institute Anticancer Drug Screen standard agent database, Bethesda, MD) with or without caspase inhibitor Z-VAD.fmk (Sigma-Aldrich). AA mimic AA005 were dissolved in 75% ethanol as a 1 mM stock solution and was kept at −80°C. MNNG (100 mM) was freshly prepared in dimethylsulfoxide (DMSO) and diluted in culture media to 0.5 mM. After treatment for 15 min, cells were washed and returned to the normal growth medium. camptothecin was dissolved in double-distilled water as a 1 mM stock solution. Z-VAD.fmk was dissolved in DMSO before use.

### Trypan blue assay

After treatments cells were harvested, resuspended in cell growth medium, and diluted 1:1 with 0.4% trypan blue stain (Sigma-Aldrich). Stained and unstained cells were counted using a hemocytometer.

### TUNEL assay

Cells were seeded in 6-well plates 1 day prior to treatments. Fragmented DNA was assessed using terminal deoxynucleotidyl transferase (TdT)-dUTP nick end-labeling (TUNEL) kit (Roche) according to the manufacturer’s protocol.

### DNA gel electrophoresis

Appropriate 10^6^ cells were harvested, and pellets were suspended in lysis buffer (0.1 M NaCl, 50 mM Tris–HCl, pH 7.5, 10 mM EDTA (ethylenedia-minetetraacetic acid), 0.5% sodium dodecyl sulfate [SDS], 500 μg/ml protease K). After a 30 minutes, incubation on ice, samples were centrifuged at 14,000 g for 30 minutes, and cellular DNA was extracted. The samples were electrophoresed in 2% agarose gel at 100 V in 40 mM Tris-acetate buffer (pH 7.4) and visualized by ethidium bromide staining.

### Flow cytometric assays for Annexin-V

Briefly, about 10^6^ cells were rinsed with phosphate-buffered saline (PBS), and Annexin-V assay was performed on a flow cytometry (Beckman Coulter) according to instructions provided by the ApoAlertAnnexin-V kit (Clontech, PaloAlto, CA) as well as stained with 50 μg/ml propidium iodide (PI; Sigma).

### RNA interference and transfection

For siRNA in SW620 cells, the following oligonucleotides were inserted into RNAi-Ready pSIREN-RetroQ vector (Clontech, Palo Alto, CA): 5′-TAGCGGTCGCCGAAATGTT-3′ (A3) and 5′-CTGGTATCCGATCAGAGAG-3′ (A5) for AIF, and 5′-ACTACCGTTGTTATAGGTG-3′ for scrambled negative control. Retrovirus with these shRNA produced in 293 T cells were used to infect SW620 cells. Stable retroviral transduction was achieved by infection for 48 h, after which selection with puromycin was initiated. Selection was stopped as soon as the non-infected control cell died off. Media were then replaced with normal growing medium.

### Western blots

The protein lysates were mixed with equal volume of Laemmli buffer (62.5 mM Tris–HCl, pH 6.8, 2% sodium dodecyl sulfate, 50 mM Dithiothreitol, 10% glycerol, 0.01% bromophenol blue), boiled for 3 min at 100°C and then resolved by sodium dodecyl sulfate–polyacrylamide gel electrophoresis on a 10%–12% gel using a mini gel apparatus (Bio-Rad, Hercules, CA). Subsequently, the proteins were electrophoretically transferred to a nitrocellulose membrane (Bio-Rad). The membranes were blocked with 5% nonfat dry milk solution in Tris-buffered saline with 0.1% Tween-20 for 1 h at room temperature and then incubated in primary antibody dissolved in block solution at 4°C overnight. The proteins were probed by antibodies against AIF (Cell Signaling, Beverly, MA), with mouse anti-β-actin mAb (Merck, Darmstadt, Germany) to confirm equal loading. After washing, the blots were incubated with horseradish peroxidase-conjugated secondary antibody (Dako Cytomation, Glostrup, Denmark) corresponding to the primary antibody in blocking buffer for 1 h at room temperature, and detections were performed by Super Signal West Pico Chemiluminescent Substrate kit (Pierce, Rockford, IL) according to the manufacture’s instructions.

### Immunofluorescence

Colorectal adenocarcinoma cells were crawled onto cover slides, fixed with 4% paraformaldehyde and permeabilized with 0.3% Triton X-100 for 10 min. Slides were blocked with 1% bovine serum albumin and incubated with rabbit anti-AIF monoclonal antibody (1:100) overnight at 4°C. After washing in PBS, the cells were stained with secondary antibodies (FITC-conjugated bovine antirabbit; 1:200; Santa Cruz Biotech) and incubated for 1 h in room temperature, followed by nuclear counterstaining with DAPI. Fluorescence signals were detected on a Olympus BX-51 fluorescence microscope (Tokyo, Japan).

### Detection of ROS by flow cytometry

Cells were washed with phosphate-buffered saline (PBS), re-suspended in pre-warmed PBS (37°C), and incubated with 10 mM 5-(and 6)-chloromethyl-2′,7′-dichlorodihydrofluorescein diacetate, acetyl ester (C-6827, CM-H2-DCFDA, Invitrogen, Carlsbad, CA, USA) for 30 min at 37°C. Cells were then washed with PBS twice and scraped into 0.3 ml of ice-cold PBS. CM-H2-DCFDA fluorescence was determined by measuring 10,000 events per sample following excitation with a 488-nm wavelength laser and reading through a 530/30 filter (FACSCalibur, BD Bioscience, San Jose, CA, USA).

### Statistical analysis

Each experiment was done independently at least 3 times with similar results. Results are expressed as mean ± S.D. Significant differences were assessed with the Student’s *t* test (2-tailed). *P* < 0.05 was considered to be significant.

## Results

### AA005 induces cell death in various cancer cell lines

**Figure 1:**
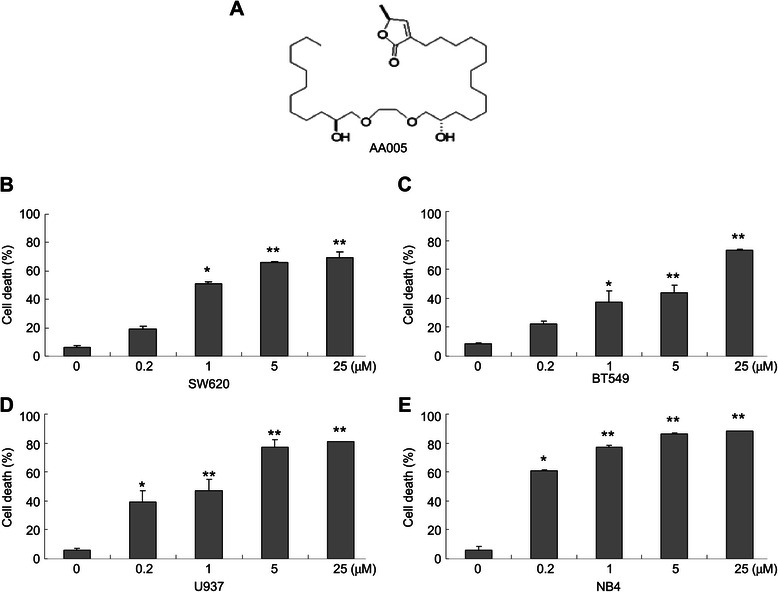
**AA005 induces cell death in various cancer cell lines. (A)** Chemical structures of annonaceous acetogenin mimic AA005. **(B–E)** Adherent cells of SW620 **(B)** and BT549 **(C)**, leukemic cells of U937 **(D)** and NB4 **(E)**, were treated with AA005 at indicated concentrations for 48 h. Cell death was measured by trypan-blue exclusion assay; each column represents the mean ± S.D. of triplicate runs in an independent experiment. *P < 0.05, **P < 0.01, compared with the vehicle-only control.

To evaluate the potential cytotoxicity of AA mimic AA005 (Figure 1A) [19], we administered AA005 to colorectal adenocarcinoma cell line SW620, breast cancer cell line BT-549, acute promyelocytic leukemia cell line NB4, and acute myelomonocytic leukemic cell line U937, followed by cell viability analysis with trypan-blue exclusion assays. Percentages of dead cells were 19.01 ± 2.10%, 50.79 ± 1.81%, 66.20 ± 0.80% and 69.55 ± 3.68% respectively, when SW620 cells were treated with 0.2 μM, 1 μM, 5 μM and 25 μM AA005 for 48 h (Figure 1B). Similar results were obtained in other cell lines (Figure 1C, D, E). These results showed that the cell-death inducing activity of AA005 was general and dose-dependent.

### AA005 induces non-canonical apoptosis

**Figure 2:**
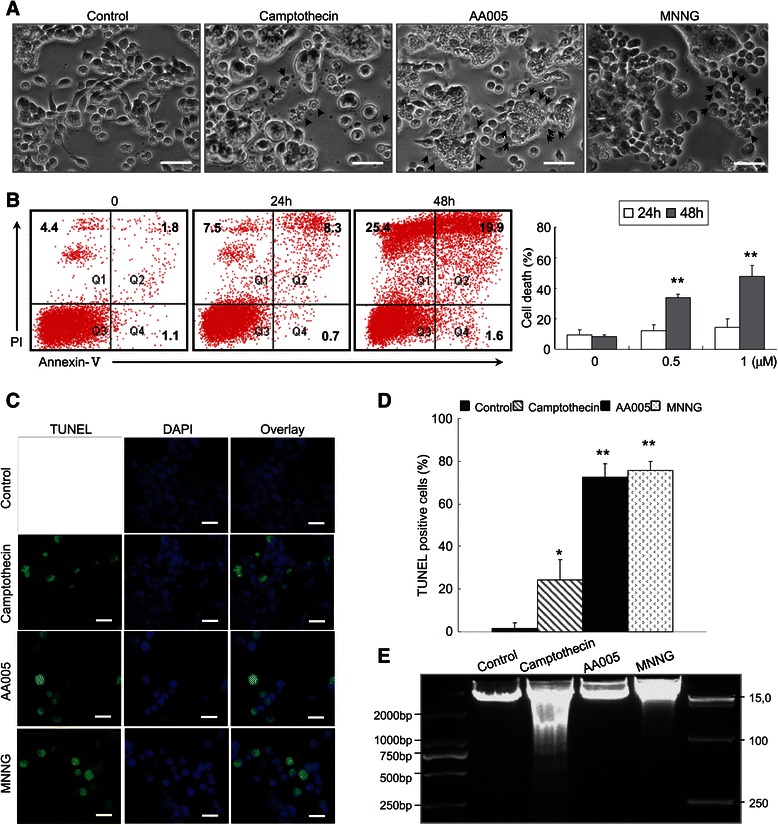
**AA005 induces non-canonical apoptotic cell death. (A)** SW620 cells were treated with or without 20 μM camptothecin, 1 μM AA005, or 500 μM MNNG, for 36 h, 48 h or 8 h. Images were viewed using an Olympus BX-51 fluorescence microscope. Treatment with MNNG and camptothecin were used as controls. Scale bars: 100 μm. **(B)** SW620 cells were treated with 1 μM AA005 for 24 h or 48 h. Annexin-V/PI double-stained cells were measured by flow cytometry. Panels show percentages of Annexin-V^+^ or PI^+^ cells. Right: dead cells. Each column represents mean ± S.D. from an independent experiment performed in triplicate. **P < 0.01, compared with vehicle-only control. **(C)** TUNEL analysis of SW620 cells treated with 20 μM camptothecin, 1 μM AA005 and 500 μM MNNG. Images were viewed using an Olympus BX-51 fluorescence microscope. Scale bars: 20 μm. **(D)** Percentages of TUNEL^+^ cells were determined in 200 cells per sample and assessed in 3 independent experiments. Results show mean ± S.D. *P < 0.05, **P < 0.01, compared with vehicle-only control. **(E)** SW620 cells were incubated with 20 μM camptothecin, 1 μM AA005 or 500 μM MNNG for 36 h, 48 h or 8 h. DNA samples from these treated cells were electrophoresed in a 2% agarose gel.

To investigate the biochemical and morphological changes during the process of AA005-induced cell death, SW620 cells were treated with AA005, N-methyl-N’-nitro-N-nitrosoguanidine (MNNG) [20], and camptothecin [21]. MNNG and camptothecin were used as controls for caspase-independent and caspase-dependent cell death, respectively. AA005-induced cell death was clearly different from camptothecin-induced cell death, which was characterized by membrane shrinking, nuclear condensation, and disintegration of the dying cell into apoptotic bodies. However, AA005-induced cell death is similar to MNNG-induced cell death during which massive cell death was triggered instantly at a certain time point, followed by the formation of cell membranes rupture, dissolution of organized structures and semi-circular shadows emerged around the dying cells (Figure 2A). Annexin-V/PI double stain-based flow cytometry analysis is the most sensitive and specific test for determining apoptotic cells, which are classified as Annexin-V^+^/PI^−^cells [22]. Although nearly 50% of cells died at 48 h of AA005 treatment, no obvious Annexin-V^+^/PI^−^cells were detected throughout this process (Figure 2B), indicating that AA005-induced cell death is not classical apoptosis. Furthermore, TUNEL analysis [23] of AA005-, camptothecin- and MNNG-treated SW620 cells are shown in Figure 2C. Although all three agents induced TUNEL^+^ cells, AA005 and MNNG showed stronger effects than camptothecin (Figure 2C, D). However, DNA fragmentation analysis indicated that camptothecin but not AA005 or MNNG induced apoptosis-specific DNA-ladders (Figure 2E). These results indicate that AA005 induced non-canonical apoptotic cell death in SW620 cells.

### AA005 induces cell death through a caspase-3 independent pathway

**Figure 3:**
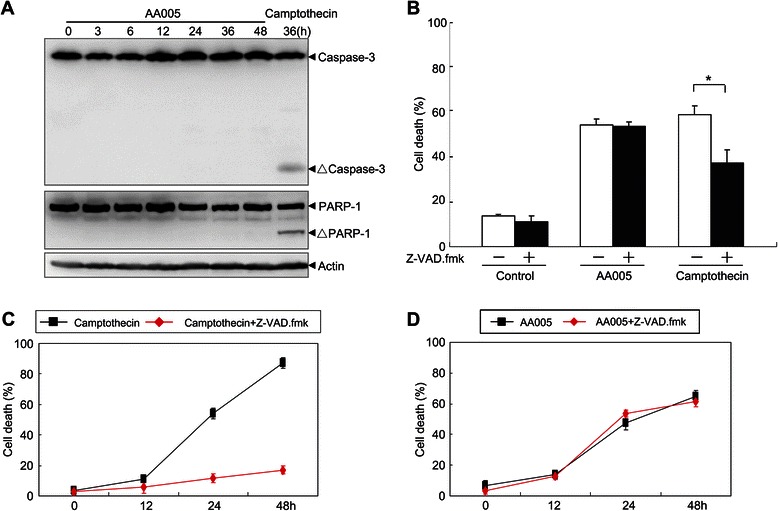
**AA005 induces cell death through caspase-3-independent mechanisms. (A)** SW620 cells were treated with or without 1 μM AA005 for the indicated times. Treatment with 20 μM camptothecin for 36 h was used as positive control. Proteins PARP-1, and caspase-3 were detected by western blot, standardized to actin. ΔPARP-1, and ΔCaspase-3 indicate cleaved 85-kDa fragment of PARP-1 and active Caspase-3 fragment proteins, respectively. **(B)** SW620 cells were preincubated with 100 μM Z-VAD.fmk for 1 h and then treated with or without 1 μM AA005 for 48 h, and with 20 μM camptothecin for 36 h. Annexin-V/PI double-stained cells and cell death were measured by flow cytometry. Results show mean ± S.D. *P < 0.05, versus camptothecin-treated group. **(C, D)** NB4 cells were preincubated with 40 μM Z-VAD.fmk for 1 h and then treated with or without 1 μM camptothecin **(C)**, or 1 μM AA005 **(D)**, for indicated times. Annexin-V/PI double-stained cells and dead cells were measured on flow cytometry. Values show mean ± S.D. of triplicates in an independent experiment.

To elucidate the mechanisms of AA005-induced cell death, we analyzed the involvement of caspase-3, an important executing caspase of apoptosis. Camptothecin treatment was used as a positive control. AA005 treatment failed to trigger cleavage of caspase-3 and its substrate PARP-1, even when cell death was as high as 55% (Figure 3A). Additionally, Z-VAD.fmk, a broad-spectrum caspase inhibitor, greatly blocked camptothecin-induced cell death, but showed no effect on AA005-induced cell death (Figure 3B). To further investigate whether caspase-3 played a role in AA005-induced cell death, we also tested caspase-3 activation in NB4 cells. Similarly, Z-VAD.fmk blocked most cell death triggered by camptothecin (Figure 3C), but had no effect on the cell death induced by AA005 in NB4 cells (Figure 3D). Taken together, these results suggested that AA005 induced cell death through caspase-3 independent mechanisms.

### AIF potentially contributes to AA005-induced cell death

**Figure 4:**
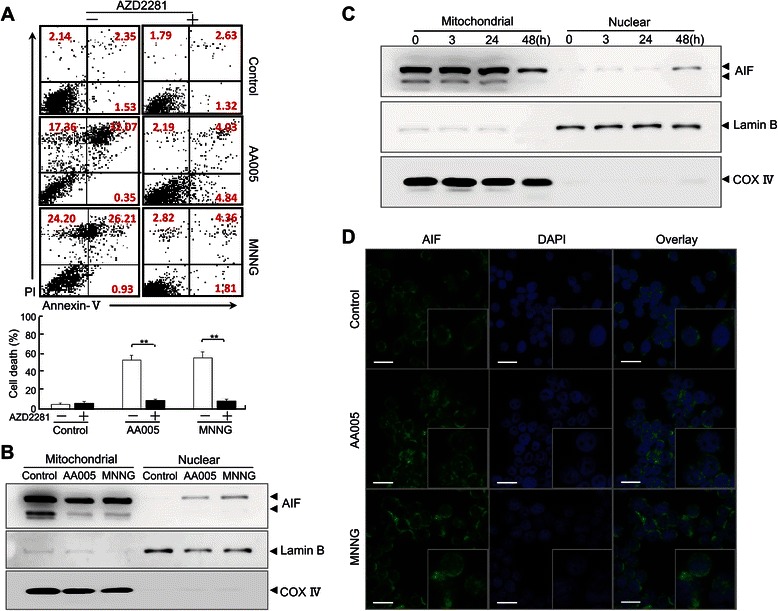
**AA005 promotes translocation of AIF to cell nuclei. (A)** Flow cytometry analysis of AA005- or MNNG-induced cell death in the presence of PARP inhibitor Olaparib (AZD2281; 50 μM). Panel numbers show percentages of Annexin-V^+^ or PI^+^ cells. Lower: dead cells. Each column shows mean ± S.D. of triplicates in an independent experiment. **P < 0.01, versus AA005- or MNNG-treated group. **(B)** Analysis of AIF translocation by subcellular fractionation. SW620 cells 48 hours after AA005 treatment, 8 hours after MNNG treatment or control cells were subjected to subcellular fractionation; immunoblotting was performed with nuclear and mitochondrial fractions. Lamin B and COX IV were used as nuclear and mitochondrial marker proteins, respectively. **(C)** NB4 cells were treated with or without 1 μM AA005 for indicated times and subjected to subcellular fractionation; immunoblotting was performed with nuclear and mitochondrial fractions. Lamin B and COX IV were used as nuclear and mitochondrial marker proteins. **(D)** SW620 cells grown on chamber slides were treated with or without 1 μM AA005 or 500 μM MNNG. Subcellular localization of AIF protein was examined by Olympus BX-51 fluorescence microscope analysis. DAPI: DAPI nuclear counterstaining. > 100 cells were inspected per experimen; cells with typical morphology are shown. Scale bars, 20 μm. The experiments in **B**–**D** were repeated at least three times with similar results.

Our data showed that AA005-induced cancer cell death left morphology similar to that of MNNG-treated cells. Reportedly, MNNG-mediated cell death was AIF-mediated and PARP-1 dependent, resulting in a caspase-independent type of apoptosis, called parthanatos [20,24,25]. Interestingly, the mitochondrial flavoprotein AIF is well known as a caspase-independent apoptosis inducer [10]. For this reason, we speculated whether AIF played a role in AA005-induced cell death. We found that inhibition of PARP by Olaparib (AZD2281) [26] in SW620 cells completely blocked AA005 or MNNG-induced toxicity (Figure 4A). Under physiological conditions, AIF resides in the inter-membrane space of mitochondria and plays an essential role in maintaining mitochondrial function [27,28]. When it is released from mitochondria and translocates to the nucleus, it induces chromatin condensation and large-scale DNA cleavage in response to death stimuli [29]. We used immunofluorescence assay and subcellular fractionation to evaluate AIF nuclear translocation. Nuclear translocation of AIF was readily observed by treatment with AA005 or the positive control MNNG in SW620 cells (Figure 4B), and similar findings in NB4 cells (Figure 4C). Immunofluorescence assays also showed AIF translocation into the nucleus after treatment with AA005 or MNNG (Figure 4D). Together, these results indicated that AA005 promotes AIF nuclear translocation.

**Figure 5:**
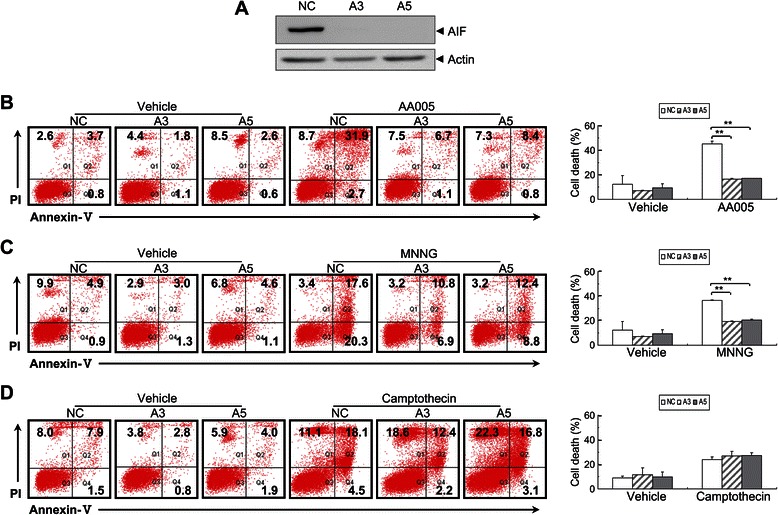
**AA005-induced cell death significantly decreases in*AIF*knockdown cells. (A)** SW620 cells were transfected with scrambled negative control (NC) or siRNAs against *AIF* (A3 or A5); absence of AIF expression was confirmed by western blot analysis, standardized to actin. **(B–D)***AIF* knockdown SW620 cells and controls were treated with or without 1 μM AA005 for 48 h **(B)**, 500 μM MNNG for 8 h **(C)**, and 20 μM camptothecin for 36 h **(D)**. Annexin-V/PI double stained cells and cell death were measured on flow cytometry. All experiments were repeated 3 times with the same results. Results show mean ± S.D. **P < 0.01, versus AA005- or MNNG-treated NC group.

To assess involvement of AIF, SW620 cells were transfected with retrovirus harboring NC or siRNAs against *AIF* (designated as A3 and A5; Figure 5A). Absence of AIF expression was confirmed by western blot analysis (Figure 5A). Furthermore, *AIF* knockdown almost completely blocked the cell death induced by AA005 (Figure 5B). We also confirmed that *AIF* knockdown inhibited the cell death induced by MNNG, the action of which is reportedly mediated by AIF (Figure 5C) [20], but had no effect on camptothecin-induced cell death, which is caspase-dependent (Figure 5D). Together, these results indicate that AA005 promote AIF nuclear translocation and trigger AIF-dependent cell death.

### ROS mediates AA005-induced cell death of SW620 cells

**Figure 6:**
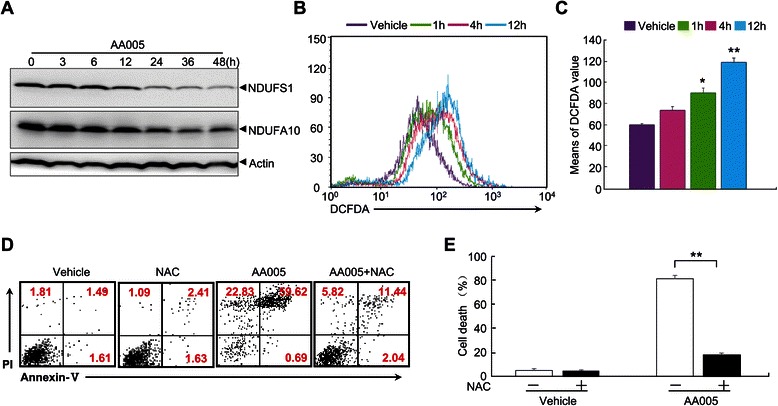
**ROS mediates AA005-induced cell death of SW620 cells. (A)** SW620 cells were treated with or without 1 μM AA005 for indicated times. Complex I subunits NDUFS1 and NDUFA10 were detected by western blot, standardized to actin. **(B−C)** SW620 cells were treated with or without AA005 at 1 μM for 1 h, 4 h or 12 h. DCFDA staining was then analyzed on flow cytometry **(B)**; quantified data: **(C)**. Experiments were repeated 3 times with the same results. Results show mean ± S.D. *P < 0.05, **P < 0.01, compared with vehicle-only control. **(D, E)** After pretreatment with or without 1 mM NAC for 2 h, cells were treated with or without AA005 at 1 μM for 60 h. Annexin-V/PI double-stained cells were measured on flow cytometry. Panels show percentages of Annexin-V^+^ or PI^+^ cells **(D)**. Dead cells **(E)**. Each column shows mean ± S.D. of triplicates in an independent experiment. **P < 0.01, vs AA005-treated group.

Because release of AIF from the mitochondria and translocation to the nucleus occurred too late during AA005-induced cell death, the intrinsic cell death signaling at the early stage initiated by AA005 should be further investigated. Evidence from other studies suggests that AAs are potent inhibitors of mitochondrial NADH-ubiquinone reductase (Complex I) [3]. Based on this clue, we tested protein levels of Complex I subunits during AA005-induced cell death. Protein levels of Complex I subunits NDUFS1 and NDUFA10 decreased significantly after AA005 treatment (Figure 6A). Increased ROS concentrations caused by depletion of mitochondrial proteins led to activation of cell death signaling [30]. As we had shown AA005 treatment to decrease mitochondrial Complex I subunits, we next investigated ROS production during AA005-induced cell death. To address whether oxidative stress contributes to responses to AA005, SW620 cells were treated with AA005 at 1 μM for 1 h, 4 h or 12 h, followed by incubation with fluorophore dichlorodihydrofluorescein diacetate (DCFDA), a dye to monitor intracellular ROS, for 30 min. As expected, intracellular concentrations of ROS were significantly increased immediately after AA005 treatment and ROS levels maintained high from 1 h to 12 h (Figure 6B, C). Importantly, elevated ROS levels were necessary to mediate AA005-induced cell death, in that N-acetyl-L-cysteine (NAC), an efficient antioxidant, largely suppressed the number of dead cells (Figure 6D, E).

### RIP1 is required for AA005-induced cell death

**Figure 7:**
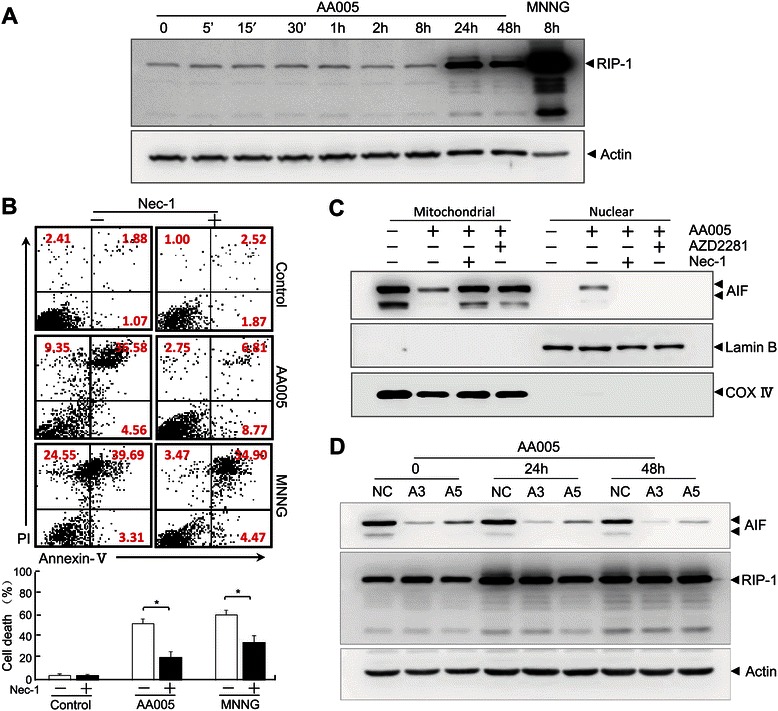
**RIP1 is required for AA005-induced cell death. (A)** Immunoblotting analysis of the expressional level of RIP-1 after 1 μM AA005 treatment or 8 h MNNG treatment for the indicated times, standardized to actin. **(B)** Flow cytometry analysis of AA005 or MNNG induced cell death in the presence of RIP-1 inhibitor Necrostatin-1 (Nec-1; 100 μM). Numbers are mean values of three independent experiments ± S.D. *P < 0.05 versus AA005- or MNNG-treated group). **(C)** Immunoblotting analysis of AIF translocation by subcellular fractionation in the presence of either RIP-1 inhibitor Necrostatin-1 (Nec-1; 100 μM) or PARP inhibitor Olaparib (AZD2281; 50 μM). Lamin B and COX IV were used as nuclear and mitochondrial marker proteins, respectively. **(D)** SW620 cells were transfected with retrovirus harboring either scrambled negative control (NC) or siRNAs against *AIF* (designated as A3 and A5). AIF and RIP-1 were examined by western blots, standardized to actin. Experiments in **(A)**, **(C)** and **(D)** were repeated at least three times with similar results.

In our study, we showed that a previously unknown caspase-independent–AIF-dependent cell death was induced by AA005, and mediated through ROS. However, the underlying mechanisms remain unclear. The present study reported that the receptor interacting protein (RIP)-1, a critical mediator of necroptosis, could modulate oxidative stress in AIF-dependent cell death [31,32]. To better understand the mechanisms, we examined the expressional level of RIP-1, and found it was up-regulated significantly in SW620 cells at 24 h with AA005 treatment (Figure 7A). We speculated that RIP-1 might participate in AA005-induced cell death. Inhibition of RIP-1 by Necrostatin-1 (Nec-1) in SW620 cells ameliorated cell death and AIF translocation after AA005 treatment (Figure 7B, C), whereas *AIF* knockdown failed to affect the increase in RIP-1 evoked by AA005 (Figure 7D). These observations imply that RIP-1 activation is required for AIF translocation from the mitochondria to the nucleus and that RIP-1 is necessary for AIF-dependent cell death induced by AA005.

## Discussion

Directed induction of cell death could provide therapeutic benefits for cancer treatment. Such treatments mainly target caspase pathways to induce apoptosis. However, caspase activation may be dispensable for some kinds of apoptosis, and increasing attention has been drawn to key molecules involved in non-apoptotic cell death or caspase-independent apoptosis [10,33]. The mitochondrial protein AIF is a new therapeutic target involved in most of the caspase-independent apoptosis systems, including programmed necrosis [34].

In this work, we found that AA005 could induce cell death of SW620 cells and NB4 cells with evidence that implies a caspase-independent mechanism; we also found that AIF might be involved in this process. We also found that AA005-induced death of cancer cells provides a morphology similar to that of MNNG treated cells. Previous studies indicated that MNNG-mediated cell death was AIF-mediated and PARP-1-dependent, resulting in a caspase-independent type of apoptosis, called parthanatos [20,25]. Our findings indicate that AA005 targets AIF signaling by promoting its nuclear translocation. The fact that AA005-induced cell death is mostly blocked by *AIF* knockdown suggests that AIF is crucial to AA005-induced cell death.

It has previously been reported that AAs were very potent inhibitors of the mitochondrial NADH-ubiquinone reductase (Complex I) and induced apoptosis by reducing intracellular cAMP and cGMP levels in human cancer [3,35]. Recently, Liu *et al.* revealed that AA005 co-localized with mitochondria in colon cancer cells. In their view, AA005 could activate AMP-activated protein kinase (AMPK) and inhibit the mTOR complex 1 (mTORC1) signal pathway, leading to growth inhibition and autophagy of colon cancer cells. However, AMPK inhibitors compound C and inosine can only partially attenuate AA005-caused proliferation suppression of colon cancer cells [36], indicating that other mechanisms affect the cancer suppression activity of AA005. In addition, the mechanism of Complex I inhibition by AA005 is unclear, although AIF is known to affect Complex I activity [24]. In fact, here we find that AA005 treatment decreased mitochondrial Complex I subunits, and significantly increased intracellular concentrations of ROS. AA005, as a novel ROS-inducing agent, may be an effective chemical probe to examine the mechanisms of tumor cells that are more sensitive and vulnerable to toxic oxidative stress [37]. Our research here shows RIP-1 activation is required for translocation of AIF from the mitochondria to the nucleus; and AIF is necessary for AA005-induced cell death, which is prevented by PARP inhibitors, RIP-1 inhibitors or knockdown of AIF, but is caspase independent. We speculate that AA005 may disrupt mitochondrial function by reducing mitochondrial Complex I expression, thus triggering ROS, RIP and AIF-dependent pathway. Thus provides a new clue to the action of AA005.

As a core executor in caspase-independent cell death, AIF is intensively studied [11]. However, many studies’ results are highly controversial. We suggest that AA005 is an effective chemical probe to examine the role of AIF. Furthermore, AA005 may be the basis of a novel treatment for cancers that are resistant to classical apoptotic reagents.

## Conclusions

AA005 can induce an AIF-dependent but caspase-independent cell death, which is mediated through ROS and RIP1. Our work shows new mechanisms for AA005-induced cancer cell death and implies a novel cancer treatment via AIF dependent cell death.

## Abbreviations

AIF: Apoptosis inducing factor
Complex I: NADH-ubiquinone reductase
MNNG: N-methyl-N’-nitro-N-nitrosoguanidine
NAC: N-acetyl-L-cysteine
Nec-1: Necrostatin-1
